# Inhibition of *Pseudomonas aeruginosa* Biofilms Using Robust Silk Fibroin-Poly(ethyleneimine) Microparticles

**DOI:** 10.3390/polym17182470

**Published:** 2025-09-12

**Authors:** Grace Neven, Tippabattini Jayaramudu, Mingyang Mao, Tugba Ozdemir

**Affiliations:** Nanoscience and Biomedical Engineering Department, South Dakota School of Mines, 501 E. St. Joseph Street, Rapid City, SD 57701, USA; grace.neven@mines.sdsmt.edu (G.N.); jayaramudu.tippabattini@sdsmt.edu (T.J.); mingyang.mao@mines.sdsmt.edu (M.M.)

**Keywords:** silk fibroin, poly(ethyleneimine), microparticles, bacteria, biofilm

## Abstract

Controlling bacterial growth and biofilm formation remains a major challenge in the treatment of chronic wounds and in preventing infection after biomedical device implantation. Thus, creating materials with inherent antibacterial potential is necessary. Here, we report silk fibroin–polyethylenimine-based (SF-PEI) microparticles to control the growth of *Pseudomonas aeruginosa*, which is a highly infectious and biofilm-forming pathogen. SF-PEI microparticles were fabricated using a solvent displacement method, and their microparticle formation was confirmed using Fourier-transform infrared spectroscopy (FTIR). The morphology and size of the microparticles were characterized using scanning electron microscopy (SEM) and dynamic light scattering (DLS). The SEM and DLS methods revealed that the microparticles formed showed a uniform, spherical morphology with a consistent size distribution, showing a Z-average of 834.82 nm. The antibacterial and biofilm inhibition properties of the SF-PEI microparticles were tested against *P. aeruginosa*. The results show significant control of bacterial growth and biofilm formation when treated with the SF-PEI particles. Further, a cell viability assay was evaluated using human dermal fibroblasts, and the results demonstrated that the SF-PEI microparticles developed demonstrated cytocompatibility, with no significant cytotoxic effects observed. These results suggest that SF-PEI microparticles offer a promising biocompatible strategy for reducing bacterial growth and their biofilm-associated infections, particularly in wound healing and medical device applications.

## 1. Introduction

Bacterial biofilms present a significant barrier to successful outcomes in both chronic wound management and implantable device performance. Biofilms are a collection of bacteria, fungi, and/or other microorganisms that adapt, communicate, and spread [1]. Bacterial biofilms grow when free-floating bacteria attach to a surface and produce an extracellular polymeric substance (EPS). In wound care, biofilms can prolong healing time and lead to further infection. Chronic and acute wounds are at risk due to the biofilm’s ability to penetrate dermal layers and disrupt the immune system. The current standard of care includes cleaning of the area, application of antimicrobials, and frequent wound debridement and dressings [2]. However, these treatments are not enough to eradicate all bacterial colonies. Biofilms in wound beds are known to regain antibiotic resistance 72 h after debridement [3]. In implants, biofilms alter the effectiveness of the device and can lead to implant removal and replacement [4]. Bacterial biofilms can be introduced during a medical procedure through direct contact, exposure to surviving bacteria on the patient, et cetera. Not only do these infections hurt the patient, but they also damage the expensive medical device itself. Current treatments include maintaining a sterilized environment, surface coating with antimicrobials, and providing long-term release of an antimicrobial agent. This current system has faults. Long-term antibiotic treatments increase antibiotic-resistant bacteria, may subject the patient to side-effects, and may damage the implant [4].

Bacterial biofilms form when free-floating bacteria attach to a surface and begin producing extracellular polymeric substances (EPSs), creating a protective matrix. This process is a significant concern in clinical settings due to its direct impact on patient health. According to the National Institutes of Health, biofilms are involved in up to 80% of microbial infections [5]. Biofilm development typically occurs in three stages: initial attachment and growth, maturation through quorum sensing, and eventual dispersal. The first step requires a suitable surface, with attachment conditions varying by bacterial species. Using biological structures such as pili or flagella, bacteria reversibly adhere to the surface, as the interactions, mainly van der Waals forces, are relatively weak at this stage. Over time, stronger adherence is established, and the bacteria begin secreting EPSs. This matrix not only anchors the cells but also offers advantages such as water retention, structural cohesion, nutrient availability, and protection from environmental threats, including antibiotics and immune responses. As the biofilm matures, quorum sensing emerges as a key regulatory mechanism. Through the release and detection of signaling molecules called autoinducers, bacteria can sense population density and coordinate gene expression accordingly. This collective behavior influences colony size, architecture, metabolic activity, and antibiotic resistance. Once the internal layers are well protected within the EPS, cells on the outer layers can detach and spread to new locations, colonizing additional surface areas.

One of the most common bacteria that creates acute and chronic infections is *Pseudomonas aeruginosa.* It is a Gram-negative bacterium that causes infections in immunocompromised individuals, such as those with implants, with cancer, post-surgery, with burns, or with HIV [6,7]. It was recognized in 2017 as a life-threatening bacterium and listed as a priority pathogen for research and development by the World Health Organization. It frequently exhibits antibiotic resistance, which is caused by its biofilm formation. The biofilm development cycle of *Pseudomonas aeruginosa* occurs in six distinct stages, each contributing to the organism’s persistence and pathogenicity. In Stage I, planktonic bacterial cells reversibly adhere to surfaces using appendages such as flagella and type IV pili [8]. Restricted flagellar movement facilitates twitching motility and the production of exopolysaccharides required for initial surface association. This attachment is material-specific, as shown by proteomic studies demonstrating that *P. aeruginosa* alters its protein expression depending on the surface. In Stage II, bacteria transition from reversible to irreversible attachment, strengthening their adhesion and initiating EPS production. Stage III involves the proliferation of attached cells into organized microcolonies. These microcolonies further develop in Stage IV into mature, three-dimensional, mushroom-like structures. The resulting architecture is shaped by bacterial motility and nutrient conditions; highly motile bacteria tend to form flat biofilms, while lower motility favors mushroom-like formations. In Stage V, cell autolysis within the biofilm’s core disrupts the matrix, creating cavities that facilitate dispersal [9]. Enzymes such as EndA degrade extracellular DNA, aiding the release of bacteria. Finally, in Stage VI, dispersed cells enter a transient state distinct from both sessile and planktonic forms, undergoing a two-hour lag phase characterized by reduced pyoverdine and c-di-GMP levels and altered gene expression [10]. These cells upregulate virulence factors and become highly cytotoxic, more sensitive to iron depletion, and significantly more virulent than their planktonic counterparts. In vivo studies have shown that dispersed cells can disseminate rapidly and cause fatal septicemia, especially following enzymatic disruption of the biofilm, underscoring their clinical importance.

Natural protein-based materials have gained increasing attention for their unique combination of mechanical strength, biocompatibility, and structural versatility, enabling a broad range of applications in biomedical and cosmetic fields. Among them, silk fibroin (SF), derived from the cocoon of the *Bombyx mori* silkworm, has emerged as a leading candidate due to its remarkable biocompatibility, tunable biodegradability, and mechanical robustness [11,12]. Composed mainly of glycine, alanine, and serine, SF features a repetitive primary sequence that favors the formation of β-sheet-rich secondary structures (Silk II), stabilized by extensive hydrogen bonding [13]. The presence of carboxyl-containing residues, such as serine, contributes to the charge distribution and conformational changes. This structural adaptability is critical for fabricating hydrogels, films, and particles. Its amphiphilic molecular architecture, characterized by β-sheet-rich hydrophobic domains and hydrophilic tails, supports diverse fabrication formats including films, hydrogels, microspheres, microneedles, and nanoparticles. During processing, SF is first degummed to remove sericin, another silk protein rich in carboxyl groups, to improve biocompatibility and allow for further manipulation. Dissolution in lithium bromide (LiBr) disrupts β-sheet interactions through ionic interference, converting the protein into a water-soluble, amorphous state that can be regenerated via dialysis. The regenerated SF can then be transformed back into more ordered β-sheet conformations through alcohol treatment, glutaraldehyde vapor annealing, or lyophilization [13]. These properties, along with its modifiability and chemical reactivity, have enabled SF to serve as a foundation for applications ranging from wound healing and cosmetic dermatology to targeted drug delivery and cancer radiotherapy [14,15]. Inspired by natural adhesion mechanisms found in marine organisms like barnacles and mussels, researchers have developed biomimetic SF-based materials incorporating catechol chemistry and metal coordination to enhance self-healing and tissue adherence [11]. When blended with Polyethylenimine (PEI), a highly cationic polymer containing primary, secondary, and tertiary amines, SF forms a stable polyelectrolyte complex driven by electrostatic attraction between the negatively charged carboxyl and carbonyl groups of SF and the positively charged amine groups of PEI [16]. Notably, branched PEI structures enable the formation of spherical microparticles capable of encapsulating bioactive agents, making SF-PEI systems highly attractive for drug delivery and antimicrobial applications. Together, the tunable structure of SF and the potent charge density of PEI form a synergistic platform for engineering multifunctional materials with tailored physicochemical and biological properties.

Recent innovations have demonstrated SF’s utility in wound care, where it has been used to support hemostasis, immune modulation, and tissue regeneration [17]. Composite formulations with chitosan and their incorporation into 3D-printed scaffolds or biosensor-integrated “smart” dressings have further expanded its clinical utility [18]. Simultaneously, SF microspheres (SFMs) have shown promise for trans arterial radioembolization (TARE) in liver cancer therapy, successfully delivering radioisotopes like ^131^I with high labeling efficiency, high biocompatibility, and sustained tumor suppression in vivo. However, translating SF into nanoscale delivery systems introduces challenges, especially regarding colloidal stability in biological fluids where ionic interactions can trigger aggregation and limit bioavailability [19]. To mitigate these effects, surface functionalization with natural and synthetic cationic polymers such as glycol chitosan, trimethyl chitosan, PEI, and PEG-PEI has been explored to enhance dispersion, cellular uptake, and circulation stability of SF nanoparticles (SFNPs) [19].

This study plans to explore the potential of SF-PEI-based materials as advanced antibacterial solutions, focusing on their biocompatibility, antimicrobial efficacy, and potential applications in medical and public health sectors.

## 2. Materials and Methods

### 2.1. Materials

Polyethylenimine branched (average Mw ~ 25,000 by LS, average Mn ~ 10,000 by GPC branched), lithium bromide (LiBr), sodium bicarbonate (NaHCO_3_), dialysis membrane, 10,000 kDa molecular weight cutoff (MWC) (Spectra/Por brand), and LB Broth Agar powder were purchased from Sigma-Aldrich (St. Louis, MO, USA). Analytical-grade acetone was obtained from Fisher Scientific (New Hampton, NH, USA). Cocoons from Bombyx mori silkworms were purchased from TTSAM (USA) via an online supplier, Amazon, and used without further modification.

### 2.2. Preparation of Silk Fibroin–Polyethylenimine (SF-PEI) Microparticles

Silk fibroin–polyethylenimine (SF-PEI) microparticles were prepared by using a solvent displacement method. Initially, silk cocoons were thoroughly washed to remove any residual silkworms. Then, the cocoons were cut into small pieces and boiled for 30 min in 2 L of reverse osmosis (RO) water containing 0.02 M sodium bicarbonate (Na_2_CO_3_) to remove the sericin. Following degumming, the obtained material was rinsed a minimum of three times with ultrapure water, with 10 min time intervals between each cycle. During this process, the unreacted material and attached residual Na_2_CO_3_ were removed. Finally, the material was dried and stored until use. In the next step, the dried fibroin was dissolved in 9.3 M of LiBr solution at 60 °C for 4 h. After obtaining the homogeneous solution, it was transferred into a dialysis membrane (10 kDa MWC) and dialyzed using ultrapure water, and the water was repeatedly changed with the following time intervals: 1, 4, 24, and 48 h.

SF-PEI microparticles were prepared by using the following methodology: 25 mL of 4% *w*/*v* silk fibroin solution was dropped into a 250 mL of acetone containing 50 mg of PEI in a 500 mL beaker. The mixture was sonicated using a Qsonica probe sonicator enclosed in a soundproof chamber for 30 min at 50% amplitude with 30 s pulses. After sonication, the solution was stirred continuously and left to dry overnight in a fume hood. The obtained microparticle suspension was lyophilized after removing the unreacted PEI and acetone, and then the lyophilized material was crushed with a mortar and pestle. A similar procedure was used to produce pure SF microparticles without using PEI for the comparison.

## 3. Characterizations

### 3.1. FTIR Analysis

Fourier-transform infrared (FTIR) spectroscopy was performed to characterize the chemical signatures of the SF and SF-PEI microparticles alongside PEI, using a Nicolet iS10 FTIR spectrometer (Thermo Fisher Scientific, Waltham, MA, USA) in the range of 4000–600 cm^−1^ with an average of sixteen scans. The raw data was then transferred into OriginPro 2019b software and plotted for peak assignments.

### 3.2. Dynamic Light Scattering (DLS) Analysis

Dynamic light scattering was used to determine the average size and polydispersity index (PDI) of SF and SF-PEI microparticles dispersed in an aqueous solution, with water as the solvent. The initial concentration was prepared at 1 mg/mL. From this stock solution, 100 µL was diluted into 10 mL, and the diluted dispersion was used for (1 mL) DLS analysis. Measurements were carried out using a Zetasizer Nano S (Model S, Serial No. MAL1000962, Malvern Instruments Ltd., Worcestershire, UK).

### 3.3. SEM and TEM Imaging

Surface morphology of the prepared SF and SF-PEI microparticles was studied using scanning electron microscopy (SEM) analysis by using a Thermo Scientific HELIOS 5CX FIB-SEM (Thermo Fisher Scientific, USA). Before performing the analysis, the microparticles were coated with carbon ~12 nm.

The size of the SF-PEI microparticles was confirmed using transmission electron microscopy (TEM) JEM-2100, equipped with a LaB6 filament operating at 200 kV (JEOL Ltd., Tokyo, Japan). For TEM analysis, a grid was prepared by depositing two to three drops of SF-PEI microparticle dispersion solution (1 mg/5 mL ethanol) onto a 3 mm copper grid. The excess solution was carefully removed with fine filter paper and allowed to dry at room temperature.

### 3.4. Antibacterial Activity Assays

Assays were performed to evaluate antibacterial activity against Gram-negative bacteria. *P. aeruginosa* (ATCC 39327) was used to evaluate the antibacterial properties of the prepared SF and SF-PEI microparticles. For this, an individual bacterial single colony strain was grown in 5 mL of LB liquid medium and incubated overnight in an orbital shaker at 37 °C. Then 100 μL of the *P. aeruginosa* bacterial solution was transferred into 5 mL of fresh, sterile LB broth. Later, 5 mg of SF and SF-PEI microparticles were added to this solution. Untreated bacterial cultures were used as a negative control to assess baseline morphology and viability. For comparison, *P. aeruginosa* was grown in plain nutrient broth without adding SF and SF-PEI microparticles. Finally, the vials were incubated at 37 °C for 24 h; after that, solution absorbance was measured using spectroscopy. This study was performed in triplicate, and average values were reported.

A live assay was performed using the SYTOX Green (Thermo Fisher) stain to assess bacterial viability. Samples were incubated with 2.50 μL of dye for 15 min at room temperature in the dark, followed by three PBS washes. Fluorescence microscopy was used to visualize stained cells.

Furthermore, scanning electron microscopy (SEM) was performed to observe bacterial morphology. Bacterial suspensions were deposited on coverslips, washed with sodium cacodylate buffer, and fixed with a Karnovsky fixative for 1 h at room temperature. Samples were then washed with distilled water, dehydrated through a graded ethanol series (35–100%), and treated with 1:1 ratio of ethanol/HMDS, followed by 100% HMDS. After air-drying and overnight desiccation, samples were mounted on aluminum stubs sputter-coated with gold, and then SEM image analysis was performed.

### 3.5. Cell Culture

Human primary dermal fibroblasts (HDFs) (PCS 201-012, ATCC) were cultured in a culture media consisting of (DMEM) (Corning, 10-013-CV, Lot 21621002), 10% fetal bovine serum (FBS) (Atlanta Biologics), and 1% penicillin–streptomycin (10,000 IU/mL, GIBCO). The cells were rinsed with warm 1X PBS to remove any traces of FBS from the surface, and a 0.25% trypsin/EDTA (1×, GIBCO) treatment was used to detach the cells from the culture flask at 37 °C for 5 min. A total of 4 mL of fresh media was added to the cells to neutralize the trypsin, and the suspended cells were removed from the flask, counted using a hemocytometer, and added to a 15 mL centrifuge tube. The cells were centrifuged for 5 min at 1500 rpms, and the supernatant was removed and replaced with a calculated amount of fresh media in which the cells were resuspended.

### 3.6. Cytotoxicity Assay

In total, 10,000 cells were seeded in each well of a 48-well plate and allowed to adhere and adjust to the culture conditions overnight. The next day, SF or SF-PEI particles were added onto the cell monolayer and incubated for 48 h. Positive controls used straight media, and negative controls were created by adding HCl to media. The day of the experiment, 10% vol/vol of PrestoBlue solution in media was added to the cell culture wells. The cells were cultured for 2 h at 37 °C with the PrestoBlue reagent before aliquots of media were withdrawn from each culture and added to a 96-well plate. A cell reader (SpectraMax i3) was used to measure the fluorescence from each culture as a measure of cell viability following the manufacturers’ recommended excitation and emission wavelengths.

### 3.7. Statistical Analysis

All data is presented as mean ± standard deviation (SD). Unless otherwise specified, the graphs depict average values, with error bars representing the standard deviation of the means, calculated from a minimum of three samples or replicates for each material. Statistical analysis was conducted using analysis of variance (ANOVA), with significance determined at a *p*-value of less than 0.05.

## 4. Results

### 4.1. FTIR Analysis

**Figure 1:**
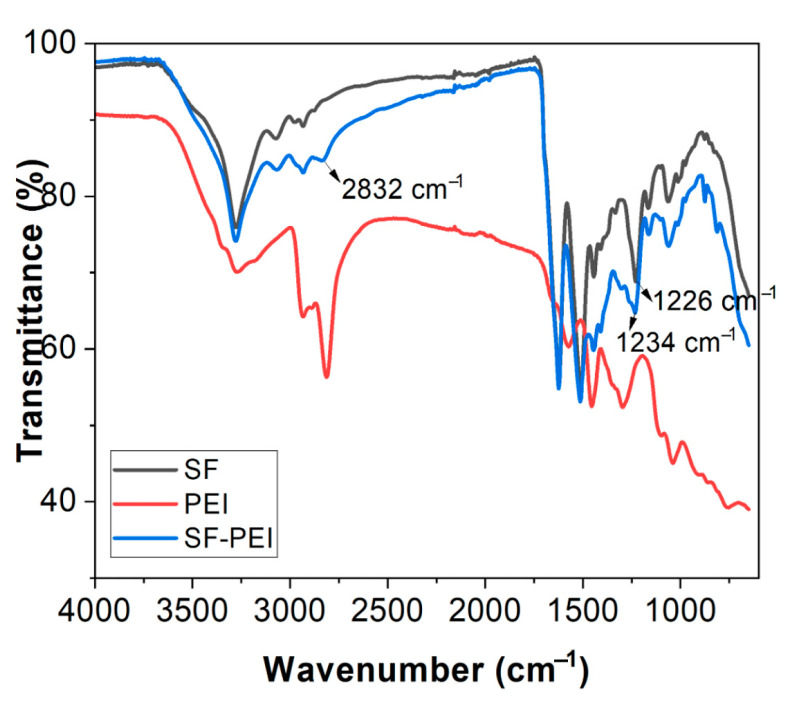
FTIR spectra of SF, PEI, and SF-PEI analysis.

FTIR analysis was used to confirm the SF-PEI microparticle formation and is a useful technique to find out the chemical functional groups and their chemical interactions. Figure 1 shows the FTIR spectra of the prepared SF microparticle (black line), showing characteristic peaks at 3277 cm^−1^ belonging to the stretching vibration of the -NH and -OH groups and peaks at 2978, 2933, and 2877 cm^−1^ corresponding to the stretching vibration of the aliphatic -CH groups. The peaks at 1621, 1510, and 1228 cm^−1^ correspond to amide I (C=O stretching), amide II (-NH bending and -C-N stretching), and amide III (-C-N stretching and -N-H deformation) [18,20]. The pure PEI (red line) shows characteristic peaks at 3272 cm^−1^ (N-H stretching of primary and secondary amines), 2933, 2888, and 2813 cm^−1^ (symmetric and asymmetric stretching of the -CH_2_ groups), 1572 cm^−1^ (N-H bending vibration of the primary amines), 1455 cm^−1^ (CH_2_ bending ), and 1296, 1097, and 1038 cm^−1^ (C-N stretching and skeletal vibrations of the PEI backbone, respectively) [21]. The SF-PEI microparticle (blue line) shows a combination of the SF and PEI peaks, with a shifting of the peaks amide I, II, and III bands (1621, 1510, and 1228 cm^−1^ to 1624, 1513, and 1234 cm^−1^). These shifting peaks suggests that the electrostatic or intermolecular interactions occurred between the functional groups of the PEI (amines) and the functional groups of the SF; these results are in good agreement with previous reports [22]. Noticeably, a new prominent peak was observed at 2832 cm^−1^ and is attributed to the C-H stretching vibration of the -CH_2_ group [23], which appeared due to the interaction of the SF and PEI molecules, as the PEI has multiple -CH_2_ and -CH_2_-NH- functional moieties in its chemical structure. The increased intensity of this band confirms the presence of PEI in the SF-PEI microparticles. Overall, the FTIR analysis confirmed the successful stabilization of SF microparticles with PEI electrostatic interaction.

### 4.2. Dynamic Light Scattering Analysis of SF and SF-PEI Microparticles

**Figure 2:**
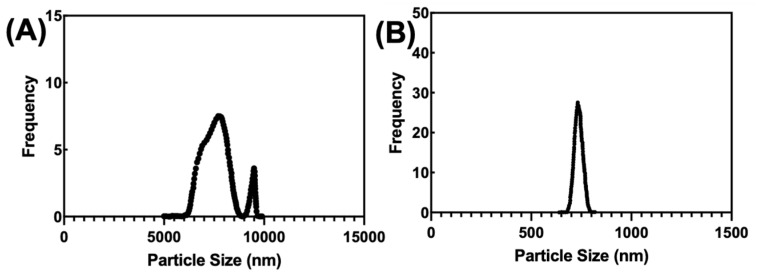
Size distributions of (**A**) SF and (**B**) SF-PEI microparticles.

The dynamic light scattering (DLS) analysis of the prepared SF and SF-PEI microparticle are shown in Figure 2. In Figure 2B, the SF-PEI microparticles demonstrate a narrow size distribution with a single sharp peak, with a Z-average (or intensity-weighted mean) of 834.82 nm. The quantitative data of Z-average size and PDI values for both SF and SF-PEI microparticles are summarized in Table 1. The sharp peak indicates that most microparticles within the sample are closely centered, reflecting a relatively homogeneous population. The Z-average accounts for larger microparticles that may dominate the light scattering signal, providing a more representative average than a simple arithmetic mean. The polydispersity index (PDI) of the SF-PEI microparticles is 0.4792, indicating a moderate degree of size variation among the SF-PEI in microparticle sizes. A PDI below 0.5 suggests that while the microparticles are not entirely monodisperse, the size distribution is fairly uniform. The narrow peak in the frequency distribution supports this conclusion. Additionally, the average microparticle size of 750.90 nm emphasizes the controlled nature of the SF-PEI microparticle preparation, whereas the frequency distribution of SF microparticles, shown in Figure 2A, reveals a broader size distribution compared to the SF-PEI microparticles. This distribution features two distinct peaks, indicating a more diverse range of microparticle sizes within the sample. The Z-average for the SF microparticles is 8043.0 nm, reflecting a bigger average microparticle size relative to the SF-PEI counterparts. The PDI of 0.407 for the SF microparticles indicates moderate polydispersity. Nevertheless, the bimodal distribution suggests two distinct size populations, implying greater heterogeneity compared to the single-peaked SF-PEI microparticles.

The calculated average microparticle size for the SF microparticles is 7977.8572±, emphasizing this diversity in microparticle dimensions. The polydispersity index (PDI) for the SF microparticles is 0.407, which is lower than that of the SF-PEI microparticles. This PDI value suggests that the size distribution of the SF microparticles is relatively narrow, indicating a more uniform size range despite the two distinct peaks present in the distribution. The lower PDI value indicates that the SF microparticles possess less variability in size compared to the SF-PEI microparticles, which is advantageous for applications requiring consistency in microparticle characteristics.

The bimodal distribution of SF particles in Figure 2A is primarily due to aggregation driven by silk fibroin’s tendency to form β-sheet-rich structures through hydrophobic interactions and hydrogen bonding. This results in two population sizes: smaller nanoparticles and larger aggregates. Other contributing factors include the preparation method, solution conditions (e.g., pH, ionic strength), and potential structural heterogeneity. In contrast, the single sharp peak in SF-PEI microparticles indicates that PEI stabilizes the particles, likely through electrostatic repulsion and surface modification, preventing aggregation.

### 4.3. Morphology Studies of Prepared Microparticles

**Figure 3:**
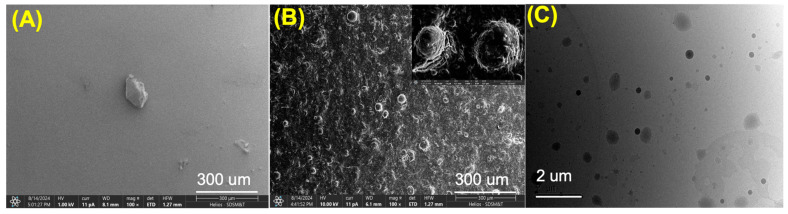
SEM images of (**A**) an SF microparticle and (**B**) an SF-PEI microparticle and (**C**) TEM images of an SF-PEI microparticle.

The morphology and microparticle size of the prepared SF and SF-PEI microparticles were analyzed using scanning electron microscopy (SEM) at 1 kilovolt and 100× magnification and transmission electron microscopy (TEM) at 5000× magnification. SEM images revealed notable differences between the SF and SF-PEI microparticles. As shown in Figure 3A, the SF microparticles exhibited larger aggregates with irregular shapes, with relatively smooth surfaces. In contrast, the SF-PEI microparticles displayed a more defined spherical morphology with a rough surface (Figure 3B). Figure 3B highlights the presence of hollow/porous spherical structures, likely resulting from PEI functionalization and subsequent self-assembly behavior. Furthermore, TEM analysis confirmed that the prepared SF-PEI microparticles have a spherical structure (Figure 3C). Based on these (SEM and TEM) analyses, the formed SF-PEI microparticles appeared spherical and well-dispersed, and the results suggest that PEI successfully interacts with SF through electrostatic interaction during formation. The distribution curve showed moderate polydispersity, suggesting that the formulation yields relatively uniform nanoparticles suitable for downstream applications such as drug delivery or gene transfection

### 4.4. Antibacterial Particles

**Figure 4:**
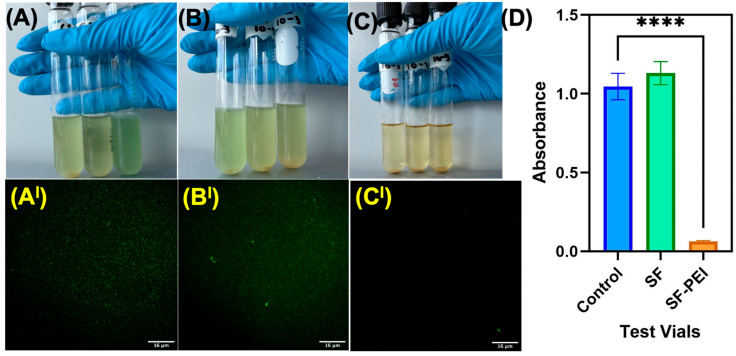
Antibacterial activity against P. aeruginosa. A–C representative images showing bacterial growth inhibition: (**A**) control, (**B**) SF microparticles, and (**C**) SF-PEI microparticles. (**D**). Absorbance after 24 h of reduction in bacterial growth with control, SF, and SF-PEI microparticles (**** indicates statistical significance at *p* < 0.0001) and Live/Dead fluorescence assay images: (**A^I^**) control, (**B^I^**) SF microparticles, and (**C^I^**) SF-PEI microparticles.

The antibacterial efficacy of SF and SF-PEI microparticles was evaluated against *Pseudomonas aeruginosa* using a broth culture assay and confirmed with a viability staining approach. After 24 h of incubation, visual inspection of the nutrient broth indicated substantial turbidity in both the control and SF-treated samples (Figure 4A,B), suggestive of bacterial growth. In contrast, the broth containing SF-PEI nanoparticles remained clear (Figure 4C), indicating significant inhibition of roughly 0.05 absorbance of bacterial proliferation. Quantitative absorbance measurements at 600 nm supported these observations (Figure 4D). Both the control and SF treatments exhibited high absorbance values, while the SF-PEI treatment demonstrated a dramatic reduction in optical density, with a mean absorbance near zero. Statistical analysis revealed this decrease to be highly significant, confirming the potent antibacterial effect of the SF-PEI formulation. Fluorescence microscopy following Live/Dead staining (Figure 4(A^I^–C^I^)) further corroborated these findings. Bacterial cells in the control and SF-treated groups showed strong green fluorescence, indicative of viable cells. Conversely, the SF-PEI group (Figure 4(C^I^)) exhibited minimal fluorescence, suggesting a marked reduction in bacterial viability consistent with cell membrane disruption or lysis. These results collectively demonstrate that while native SF exhibits negligible antibacterial activity, its combination with PEI imparts potent bactericidal properties to the particles, making SF-PEI microparticles a promising candidate for antimicrobial applications.

**Figure 5:**
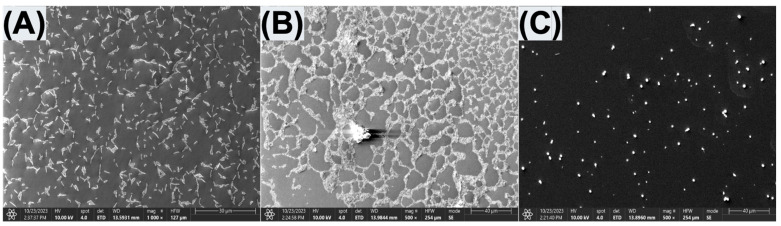
SEM surface morphology images of bacteria: (**A**) control, (**B**) SF microparticle, and (**C**) SF-PEI microparticles at 10 kilovolts and 1000 magnification.

The SEM images in Figure 5 provide a detailed view of the surface morphology and bacterial presence for the control, SF, and SF-PEI-treated samples. In the control sample (Figure 5A), there is a dense presence of bacteria, with no visible particles on the surface. The bacteria are uniformly distributed across the sample, indicating a healthy bacterial population in the absence of any antimicrobial treatment or microparticles. The SF-treated sample (Figure 5B) shows both bacteria and SF microparticles co-existing on the surface. Despite the presence of SF microparticles, the bacterial abundance remains comparable to the control, suggesting that the SF particles do not exert a bactericidal effect. The SEM image shows a combination of particles and bacteria scattered across the surface, further confirming the inert nature of SF with respect to bacterial viability. In contrast, the SF-PEI-treated sample (Figure 5C) shows a complete absence of bacteria, with only SF-PEI microparticles visible on the surface. The lack of bacterial presence suggests a potent antimicrobial effect of the SF-PEI microparticles, likely due to the PEI component. The SEM image demonstrates a clear reduction in bacterial colonization, with a sparsity of biological material and a marked increase in particle density. Overall, the SEM results corroborate the findings of the Live/Dead Assay, showing that while SF microparticles do not affect bacterial survival, SF-PEI microparticles effectively eliminate bacterial presence on the surface.

### 4.5. Cell Viability Assay

**Figure 6:**
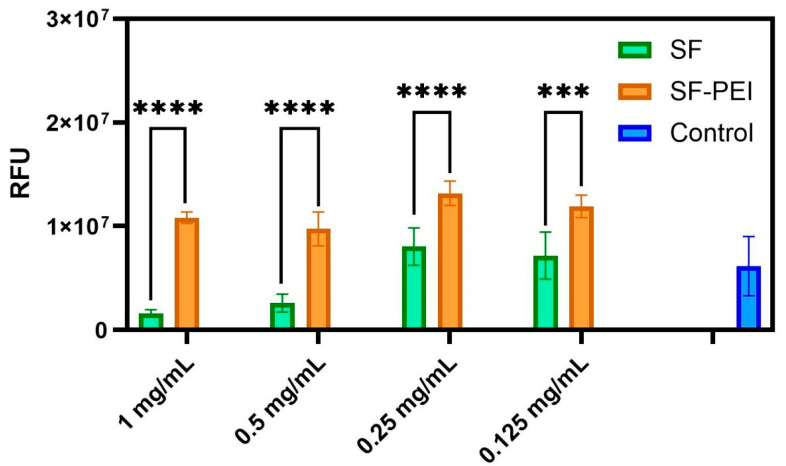
Cell viability of human dermal fibroblasts (HDFs) after treatment with SF and SF-PEI at various concentrations (1, 0.5, 0.25, and 0.125 mg/mL) (*** indicates statistical significance at *p* < 0.001 and **** indicates statistical significance at *p* < 0.0001).

Cell viability was assessed using the PrestoBlue assay, and the results are presented as relative fluorescence units (RFUs) (Figure 6). Compared to the control, SF (1 mg/mL) alone did not significantly increase cell viability. However, all formulations containing polyethyleneimine (PEI) showed significantly enhanced RFU values, indicating increased metabolic activity. Notably, the SF-PEI (0.5 mg/mL) group exhibited the highest RFU among all tested conditions, suggesting a strong synergistic effect between silk fibroin (SF) and PEI at this concentration. Specifically, the SF-PEI group showed a 3.86-fold increase over SF alone and a 1.58-fold increase over the control at 0.5 mg/mL. At 1 mg/mL, SF-PEI demonstrated a 6.68-fold increase compared to SF and a 1.76-fold increase over the control. At 0.25 mg/mL, the fold increases were 1.64-fold over SF and 2.14-fold over the control, while at 0.125 mg/mL, they were 1.66-fold and 1.94-fold, respectively. The SF-PEI (0.125 mg/mL) and PEI-only (0.125 mg/mL) groups also demonstrated significantly higher viability compared to SF or control groups, though to a lesser extent than the higher PEI concentrations. Overall, these findings suggest that the incorporation of PEI, particularly at 0.5 mg/mL, substantially promotes cell metabolic activity when combined with SF.

## 5. Discussion

The history of wound care spans thousands of years, with early civilizations such as the Egyptians pioneering the use of natural antimicrobial agents and primitive dressings. They applied honey to wounds for its healing properties and crafted plasters using a mixture of honey, grease, and lint [24]. Remarkably, they also used green pigment containing copper, now known to be toxic to bacteria, to coat wounds, as a form of infection control. Today, wound care has evolved into a highly advanced field, with more than 5000 products available that incorporate materials designed to be highly absorbent and promote optimal healing environments. Despite this progress, infections at wound sites, particularly chronic infections, remain a major challenge. These infections can significantly delay healing and place a substantial burden on healthcare systems [25]. A key contributor to this problem is the formation of biofilms—complex microbial communities encased in a self-produced matrix that adheres to tissue and protects bacteria from antibiotics, biocides, and immune responses. Compounding this issue is the global rise in antimicrobial resistance, which renders many conventional treatments ineffective [26]. To address these challenges, recent advances in nanotechnology have introduced novel strategies for enhancing wound care. Metal nanoparticles, particularly silver nanoparticles, are widely recognized for their broad-spectrum antimicrobial activity against bacteria, fungi, yeast, and viruses [27]. These particles are often incorporated into hydrogels and foam-based dressings to provide sustained antimicrobial effects. However, silver nanoparticles tend to aggregate, requiring immobilization on suitable substrates to maintain their effectiveness and ensure controlled delivery [27]. Moreover, concerns are emerging about the development of resistance to silver, as well as its potential cytotoxicity to human cells [26,28]. In response, researchers are increasingly exploring bio-inspired alternatives that combine natural materials with nanotechnology. Silk fibroin, a fibrous protein known for its structural integrity and self-assembling properties, has shown significant promise as a scaffold for incorporating antimicrobial agents like silver nanoparticles or quantum dots [12,29]. These silk-based composites offer both antimicrobial activity and improved biocompatibility, making them compelling candidates for the next generation of advanced wound dressings.

Beyond antimicrobial applications, silk fibroin (SF), the core structural protein derived from *Bombyx mori* cocoons, has emerged as a versatile and sustainable biopolymer for the development of nanoparticulate and microparticulate drug delivery systems [30]. Its appeal lies in its biocompatibility, controlled biodegradability, mechanical robustness, and unique ability to undergo conformational transitions that allow for fine-tuning of material properties [31]. Unlike spider silk, which consists mainly of spidroins, silkworm silk comprises heavy and light fibroin chains surrounded by sericin proteins. Sericin must be removed through a degumming process to yield purified silk fibroin suitable for biomedical use [32,33]. Otherwise, an immunogenic response may occur in vivo [34]. Once degummed, fibroin can be solubilized in chaotropic salt solutions such as lithium bromide and regenerated into aqueous form, enabling downstream fabrication into diverse morphologies including nanoparticles, microparticles, films, scaffolds, and hydrogels [35]. Among these, nanoparticulate and microparticulate formats are particularly attractive for drug and gene delivery due to their high surface area, tunable degradation rates, and ability to encapsulate both hydrophilic and hydrophobic compounds [36].

A clear demonstration of SF’s versatility is seen in the successful fabrication of SF-PEI microparticles using a solvent displacement method. The process involved degumming raw silk cocoons, dissolving the fibroin in a chaotropic solution, and dialyzing it to produce an aqueous SF solution. This solution was then added dropwise into an acetone solution containing PEI under sonication and continuous stirring. Following solvent evaporation, the resulting particles were ground into a dry powder. This simple and scalable method produced functionalized SF-based particles with potential for gene delivery and other biomedical applications.

A key advantage of SF in such systems lies in its ability to transition from the metastable Silk I conformation in random coils and helices to the highly stable, β-sheet-dominated Silk II structure [36]. This transformation, triggered by changes in pH, temperature, ionic strength, or mechanical shear, significantly affects the physicochemical properties of SF particles, including stability, drug release kinetics, and mechanical strength. β-sheet-enriched particles exhibit enhanced structural integrity and slower degradation, making them ideal for sustained-release applications [31]. Electro gelled silk, another SF processing method involving electric fields, offers additional customization, with amorphous e-gels allowing selective crosslinking to control degradation and enable on-demand drug or cell release. Taken together, these features underscore SF’s value as a multifunctional carrier system with exceptional tunability. Coupled with its widespread availability from established sericulture practices and its strong biocompatibility profile, silk fibroin continues to stand out as a leading platform for next-generation nano- and microparticulate delivery systems.

This becomes particularly significant in the context of combating multidrug resistant pathogens, such as *Pseudomonas aeruginosa*, which is a widespread Gram-negative bacterium. It is responsible for a range of hospital-acquired infections and poses a significant threat to immunocompromised individuals, including those with cancer, recent surgeries, severe burns, or HIV infection [6,37]. In 2017, the World Health Organization identified *P. aeruginosa* as one of the most dangerous pathogens and classified it as a priority target for antibiotic research and development [6]. Misidentification of *P. aeruginosa* infections remains a persistent and growing concern in clinical settings, largely due to the bacterium’s ability to adopt diverse morphologies, including small-colony variants, rough variants, and wrinkled forms. In addition, recent findings suggest that injured fascia can actively trigger *P. aeruginosa* virulence, revealing a host–pathogen interaction where tissue damage itself enhances bacterial aggression [38]. This dynamic activation of virulence through quorum sensing and iron-scavenging systems may explain the persistence of infection [38]. As infections advance and transition into chronic stages characterized by biofilm formation, *P. aeruginosa* isolates exhibit distinct phenotypes, often appearing as cell aggregates that form adhesive or “sticky” colonies [6]. Notably, biofilm-associated bacteria from long-standing chronic infections, such as those found in the lungs of cystic fibrosis patients, often display extremely slow growth or become non-culturable [6]. This presents a major challenge for traditional diagnostic methods, as conventional microbial culture and biochemical assays frequently fail to detect these biofilm-embedded forms [39,40]. Most clinical laboratories in the U.S. rely on commercial automated systems for antimicrobial susceptibility testing (AST), such as the bioMérieux Vitek 2 [29]. However, multiple studies have reported that these systems may fail to accurately detect resistance in Gram-negative bacteria species like *Pseudomonas aeruginosa* [29]. *P. aeruginosa* is a major contributor to chronic wound infections, largely due to its exceptional ability to form biofilms [6]. The persistence of *P. aeruginosa* in infected wounds is primarily driven by its secretion of extracellular polymeric substances (EPSs), including exopolysaccharides, matrix proteins, and extracellular DNA (eDNA) [41,42]. These components collectively encapsulate bacterial cells and form a robust matrix [33]. Biofilms firmly attach to surfaces or tissues, creating a protective environment that enhances bacterial survival [43]. In *P. aeruginosa*, the biofilm matrix not only provides structural integrity but also enhances antimicrobial resistance, due to the bacterium’s inherently low outer membrane permeability and highly adaptive resistance mechanisms. Moreover, the ability of *P. aeruginosa* to colonize the surfaces of medical devices further complicates clinical management, as biofilm formation on these devices often leads to persistent and hard-to-eradicate infections. Consequently, *P. aeruginosa* represents a critical challenge in both wound care and medical device safety due to its biofilm-forming capacity and resistance profile. Given these challenges, it is essential to develop materials that can actively inhibit bacterial growth and biofilm development. Our data demonstrate that SF-PEI particles meet this need effectively. The SEM images in Figure 3 provide clear evidence that, unlike untreated and SF-only surfaces, which remain heavily colonized by bacteria, SF-PEI-treated samples show a complete absence of bacterial presence. Where SF alone fails to limit microbial survival, the incorporation of PEI results in a surface hostile to *P. aeruginosa*. This outcome strongly supports the role of SF-PEI as a surface-active antimicrobial agent. The images, together with the Live/Dead assay, provide a compelling case that SF-PEI particles can prevent both bacterial adhesion and biofilm formation—key strategies in overcoming the persistence and resistance that make *P. aeruginosa* so difficult to eliminate.

To further validate this effect, additional quantitative and morphological assessment were conducted, revealing that SF-PEI particles demonstrated significant antibacterial properties in both absorbance-based assays and SEM analysis, effectively eliminating *Pseudomonas aeruginosa* growth in treated samples. This activity is attributed to the Polyethylenimine (PEI) component, a highly cationic polymer [44]. It has a high density of primary, secondary, and tertiary amine groups that give it a strong positive charge [16,45]. Its availability in a variety of molecular weights and structural forms determines its bactericidal action [46]. It is known for disrupting bacterial cell wall integrity and inhibiting biofilm formation [47,48]. The antibacterial activity of PEI is likely driven by osmotic swelling that occurs after its uptake into the cell [49]. As an effective proton sponge, PEI causes an influx of protons and counterions into endosomal compartments, leading to polymer expansion and subsequent disruption of the endosomal membrane. This enables drugs or polymers to enter the cytosol more effectively and carry out their intended functions. Furthermore, the quaternary ammonium groups in PEI contribute to its antimicrobial effects by interacting with and disrupting the negatively charged bacterial cell membrane [39,49]. This membrane damage results in the leakage of intracellular contents, including potassium ions, ultimately leading to rapid bacterial cell death [49]. The absence of viable bacteria in SF-PEI-treated samples, as shown in Figure 5, highlights the effectiveness of this antibacterial mechanism. The particle size of SF-PEI offers a notable advantage, as the high surface area-to-volume ratio enables a greater concentration of active antibacterial groups at the interface, enhancing performance even at low doses [50]. Building on these promising findings, future studies should investigate the antibacterial spectrum of SF-PEI particles against additional bacterial strains, including Gram-positive species, and assess the long-term stability and release kinetics of the PEI coating under physiological conditions. Such insights are critical for advancing SF-PEI applications in clinical contexts such as wound dressings or implant coatings.

However, translating these antimicrobial benefits into clinically viable wound care solutions requires careful consideration of host tissue compatibility. While the use of antibacterial agents is essential for preventing infection and promoting timely healing, an ideal wound dressing must strike a careful balance between antimicrobial efficacy and the preservation of host cellular function. Successful tissue regeneration depends on the survival, proliferation, and spatial organization of cells within the scaffold [51]. Studies have shown that inadequate cell density and poor vascularization can compromise the regenerative potential of silk fibroin scaffolds when seeded [42]. When considering wound dressing, it is especially critical to avoid cytotoxic effects on resident dermal fibroblast cells, which are vital to wound repair [52]. Fibroblasts are key producers of extracellular matrix (ECM) that provide structural support, mechanical resilience, and biochemical signals essential for organ function and tissue integrity [53]. Through ECM organization and secretion of cytokines and growth factors, they regulate the local microenvironment and guide neighboring cell behavior [53,54]. Additionally, fibroblasts can act as progenitors for specialized mesenchymal lineages, including osteoblasts and adipocytes, during development, homeostasis, and tissue repair [53]. Recruited to the wound site within 5–7 days after injury, fibroblasts synthesize and deposit the extracellular matrix (ECM) to restore the skin’s structural framework and, in later stages, facilitate collagen remodeling to strengthen the newly formed tissue [45]. However, chronic inflammation in the wound microenvironment driven by oxidative stress, impaired angiogenesis, elevated levels of proinflammatory cytokines, and bacterial infection can lead fibroblasts to enter a senescent state [52]. These senescent-like fibroblasts are less capable of recruiting neutrophils, which compromises the immune response and facilitates the formation of biofilms [55]. In this context, the development of biocompatible antibacterial materials and preserving fibroblast viability is of great importance. The cell viability assays have demonstrated that silk fibroin–polyethylenimine (SF-PEI) composites significantly enhance cell viability compared to SF alone and HDF controls, as demonstrated by the obvious increase in metabolic activity shown in Figure 6. This highlights SF-PEI’s potential for tissue engineering applications. This result underscores SF-PEI’s favorable biocompatibility, making it a promising candidate for multifunctional wound dressings that not only combat infection but also support cellular processes essential to healing.

## 6. Conclusions

In this study, we successfully fabricated and characterized silk fibroin–polyethylenimine (SF–PEI) microparticles, demonstrating their multifunctionality for potential biomedical applications. FTIR analysis confirmed the chemical conjugation between silk fibroin and PEI, as evidenced by a shift in the amide III band and enhanced C-H stretching peaks, indicating incorporation of aliphatic and amine-rich moieties from PEI. DLS results revealed that SF-PEI microparticles exhibited a more homogeneous size distribution compared to native SF particles, with a sharp unimodal peak and moderate polydispersity, suggesting effective stabilization by PEI. Morphological analysis via SEM and TEM further supported these findings, showing spherical, porous structures unique to the SF-PEI conjugates. Functionally, SF-PEI particles exhibited potent antibacterial activity against *Pseudomonas aeruginosa*, significantly reducing both planktonic growth and surface colonization, as confirmed by bacterial culture assays, Live/Dead staining, and SEM imaging. Importantly, PrestoBlue cell viability assays showed that SF-PEI particles not only preserved but significantly enhanced fibroblast metabolic activity compared to SF alone, highlighting their cytocompatibility. Together, these findings underscore the promise of SF-PEI microparticles as dual-function wound-healing materials capable of both preventing infection and supporting tissue regeneration.

## Figures and Tables

**Table 1:** Z-average and polydispersity index (PDI) values from dynamic light scattering (DLS) measurements.

	SF-PEI Microparticle	SF Microparticle
Z-Average	834.82 nm	8043.0 nm
Polydispersity	0.4792	0.407

## Data Availability

The original contributions presented in this study are included in the article; further inquiries can be directed to the corresponding author.
